# Reference equation for measurement of the maximal dynamic inspiratory muscle pressure index (S-Index) in healthy Brazilian adults

**DOI:** 10.36416/1806-3756/e20240312

**Published:** 2025-12-11

**Authors:** Luis Felipe da Fonseca Reis, Cleber da Penha, Pamela do Carmo Dosso da Silva, Aline Oliveira Martins Soares de Mendonça, Ana Carolina Sebastião da Silva, Clara Pinto Diniz, Flavia Mazzoli-Rocha, Arthur de Sá Ferreira, Agnaldo José Lopes

**Affiliations:** 1. Programa de Pós-Graduação em Ciências da Reabilitação, Centro Universitário Augusto Motta, Rio de Janeiro (RJ), Brasil.; 2. Instituto Nacional de Infectologia Evandro Chagas, Fundação Oswaldo Cruz, Rio de Janeiro (RJ), Brasil.; 3. Programa de Pós-Graduação em Ciências Médicas, Faculdade de Ciências Médicas, Universidade do Estado do Rio de Janeiro, Rio de Janeiro (RJ), Brasil.

**Keywords:** Respiratory muscle strength, S-Index, Respiratory muscle assessment

## Abstract

**Objectives:**

Several equations for calculating maximal inspiratory pressure (MIP) have been validated for the Brazilian population; however, none exist for maximal dynamic inspiratory muscle pressure (S-Index).

**Methods:**

This cross-sectional study was conducted at two centers following approval by the institutional ethics committee. Healthy Brazilian adults were sequentially randomized to assess either the MIP or S-Index. Pulmonary function (spirometry), peripheral muscle strength (handgrip strength of the dominant upper limb - HGdUL), and physical activity level (IPAQ) were also evaluated. The S-Index and MIP values were reported as absolute values and compared using the Wilcoxon paired test. Multiple linear regression was used to develop reference equations. Lower limits of normality (LLNs) were stratified by sex and age using Z-scores, providing cut-off points to define inspiratory muscle weakness via the S-Index Deviation Score (SDS).

**Results:**

The final sample comprised 214 eutrophic volunteers, 50% men, with a mean age of 43.1 ± 15.0 years. The median MIP was significantly higher than the median S-Index (97.2 [96.7-112.0] vs. 92.5 [80.0-105.0] cmH_2_O; p<0.001). The predicted equation for the S-Index, which used age, sex, and HGdUL as predictors, was: S-Index = 69.72 + 10.765×sex (men = 1; women = 0) - 0.211×age + 0.797×HGdUL. Additionally, the LLNs and cut-off points for ventilatory muscle weakness by sex and age group were established.

**Conclusions:**

This study provides the first reference values for the S-Index in healthy, eutrophic Brazilian adults, including LLNs and cut-off points for diagnosing ventilatory muscle weakness.

## INTRODUCTION

Maximal inspiratory pressure (MIP) and maximal expiratory pressure (MEP) are widely used in clinical practice because of their reliability and accessibility.^1^ These measurements assess ventilatory muscle strength under static conditions,^2^ ^,^ ^3^ in contrast to the dynamic contractions observed in physiological states. Conditions such as chronic obstructive pulmonary disease (COPD), asthma, obesity, and neuromuscular disorders can impair ventilatory muscle dynamics by altering the length-tension relationship, leading to dysfunction.^3^

In order to evaluate inspiratory muscle strength under dynamic conditions, researchers generally use a portable electronic device equipped with a gate valve and variable flow control, initiating the measurement at residual volume. Flow and pressure signals are typically sampled at 500 Hz to calculate the maximal dynamic inspiratory muscle pressure, known as the S-Index.^4^ ^,^ ^5^ The S-Index represents the peak inspiratory pressure at the highest point on the pressure-lung volume curve. As with other isokinetic devices, a minimum load of 3 cmH_2_O is applied to create resistance to airflow, enabling detection of flow variations. A mathematical algorithm is then used to calculate the S-Index.^6^ ^,^ ^7^ This approach allows the identification of variations in airflow and lung volume, assisting in the interpretation of inspiratory muscle weakness.^5^ ^-^ ^8^

This device has since been increasingly used in Brazil and other countries for the clinical screening of both acute and chronic conditions.^9^ ^-^ ^14^ However, due to the lack of established reference values for the S-Index, clinicians often rely on MIP measurements and reference equations to estimate this parameter. Nevertheless, the MIP does not accurately reflect dynamic inspiratory performance. 

Therefore, the aim of the present study was to develop a predictive equation for the S-Index in healthy Brazilian adults and to establish its LLNs and criteria for diagnosing ventilatory muscle weakness.

## METHODS

This study proposes a new equation for predicting the S-index in healthy Brazilian adults, establishing LLNs and diagnostic criteria for ventilatory muscle weakness. During a single outpatient visit, sociodemographic data, medical history, and smoking status were obtained through structured interviews carried out between December 2022 and November 2023. 

Adult volunteers residing in Rio de Janeiro, southeastern Brazil-but originally from 12 different Brazilian states-were recruited via public invitations posted on social media. The participants were matched by sex and age (range: 20-65 years), were non-smokers, and had normal weight (body mass index [BMI]: 25.45 ± 3.31 kg/m^2^). Exclusion criteria included a history of respiratory, cardiovascular, or neuromuscular disease; spirometric abnormalities at baseline; difficulty understanding test instructions; or significant pain/discomfort during the evaluation. 

Height and weight were measured using a digital scale (precision: 0.1 kg) attached to a stadiometer (accuracy: 0.005 m), and BMI was determined. Physical activity level was assessed using the short version of the International Physical Activity Questionnaire (IPAQ), validated in Portuguese, and was categorized as low (<600 MET-min/week), moderate (600-3,000 MET-min/week), or high (>3,000 MET-min/week).^16^

Handgrip strength was assessed using a Jamar 90 kg/200 lb hydraulic handgrip dynamometer (JLW Instruments, Chicago, IL, USA), which has a measurement range of 0.5-90 kg and a resolution of 0.05 kg. Participants were seated with the arm positioned parallel to the body, elbow flexed at 90°, and their forearm and wrist were in a neutral position. Three measurements were taken for each upper limb, alternating between the dominant and non-dominant hands, with a 1-minute rest interval between attempts. The highest value from each hand was recorded. 

Pulmonary function was evaluated using a computerized spirometry system (Koko SX 1000, nSpire Health, USA), following standard protocols to measure forced expiratory volume in the first second (FEV_1_), forced vital capacity (FVC), and the FEV_1_/FVC ratio. The results were then compared with the predicted values for the Brazilian adult population as described by Pereira et al. (2007).^19^

All participants were randomly assigned to the sequence of assessment procedures using a six-sided die. Rolls of 1, 2, or 3 indicated that the participant would begin with static maximal respiratory pressure measurements (MIP and MEP) first, followed by dynamic maneuvers, while rolls of 4, 5, or 6 began with dynamic inspiratory muscle pressure (S-Index) measurements followed by static maneuvers.

The MIP and MEP were determined using a digital vacuum manometer (MVD 300, Globalmed, Porto Alegre, RS, Brazil), following ATS/ERS guidelines.^1^ MIP was measured from residual volume with closed airways, while MEP was measured from total lung capacity. Pressure was recorded after sustaining effort for at least 3 seconds, and the plateau value was registered as the MIP or MEP. A minimum of five acceptable maneuvers was performed, with at least three showing less than 10% variability. The highest value from the three reproducible maneuvers was used and expressed both as an absolute value and as a percentage of the predicted value based on the equation by Neder et al. (1999).^20^

The maximal dynamic inspiratory muscle pressure (S-Index) was measured using a Powerbreathe K5^®^ device and analyzed with Breathelink^®^ K5 software, version 2.1.1. This is currently the only available device capable of performing such measurements. It is flow-oriented and electronically controlled, and estimates the S-Index by integrating peak inspiratory flow and volume over time. The device contains a valve that adjusts its diameter in response to inspiratory flow and calculates muscle strength with the airway open. 

The measurements were obtained after the participants performed maximal, rapid inspiratory efforts through a properly fitted mouthpiece, with the valve open, under verbal encouragement from the evaluator. Prior to testing, each participant completed 10 unmeasured moderate-intensity warm-up maneuvers, followed by 8 maximal-effort maneuvers, of which at least 3 had to be acceptable. A minimum 1-minute rest interval was allowed between each maximal effort to avoid muscle fatigue. The S-Index varied by less than 10% among the three acceptable maneuvers, and the highest peak value was recorded. 

All evaluations at the two participating centers followed the same protocol. Eight evaluators-specialists in the field-received three months of training from the lead researchers before data collection began. 

This study was approved by the Institutional Ethics Committee of the coordinating center, registered on the Brazil Platform (CAAE No. 64320022.4.000.5235). All participants provided written informed consent.

### Statistical analysis

The sample size was calculated using the equation proposed by Tabachnick and Fidell (1983): N > 50 + 8 K, where K represents the number of independent variables.^22^ This study included six independent variables: sex, age, weight, height, handgrip strength, and physical activity level (assessed using the IPAQ). Accounting for a 20% loss, the required sample consisted of at least 100 participants of each sex. The volunteers were matched by sex and age, resulting in a total of 250 healthy adults (125 men, 125 women), distributed across five age ranges for each sex: 20-29, 30-39, 40-49, 50-59, and 60-65 years. 

Multiple linear regression analysis was conducted using the *enter* method, whereby each variable hypothesized to influence the S-Index was added in successive steps. ANOVA was used to determine whether each variable significantly improved the model’s predictive accuracy for the S-Index. 

Sex-specific reference equations were developed using multiple linear regression models. The associations between MIP, S-Index, and other relevant variables were analyzed to assess the dependence of S-Index and MIP on categorical variables. For continuous variables, either the t-test or the Mann-Whitney U test was applied to evaluate the strength of linear dependence, and outliers were identified. 

Two regression models were constructed: one with sex and age as independent variables, and a second model incorporating sex, age, and the HGdUL measurement. All regression models were evaluated for compliance with standard assumptions, including absence of multicollinearity among independent variables, independence of residuals, absence of outliers, normally distributed residuals, homoscedasticity, and linearity between dependent and independent variables.

ANOVA testing was performed between the two nested models to select the best predictive model and establish the most appropriate equation. The LLN of the S-Index in men and women across the age groups was calculated using Z-scores. A Bland-Altman analysis was conducted to assess the agreement between the values ​​predicted by the model’s equation and the actual S-Index measurements.

### Lower limit of normality (LLN)

The 5th and 95th percentile limits of a healthy population can be used to identify individuals with unusually low or high results, respectively.^23^ These percentiles are based on the reference interval, which reflects the distribution of expected values in a healthy population. The LLN serves as a cut-off to define results falling outside the typical range observed in clinical practice. 

For the S-Index, the LLN for men and women in each age group was calculated using Z-scores, defined as values ≥1.645 standard deviations below the group mean. Z-scores or population-based percentage values describe the probability of a given result occurring within the distribution of healthy individuals. In spirometry, the 5th percentile (corresponding to a Z-score of -1.645) is commonly used as a threshold for low values, acknowledging a 5% false-positive rate among healthy individuals. 

The S-Index Deviation Score (SDS) indicates how many standard deviations a value falls below the peak mean S-Index, providing a descriptive and context-appropriate metric for identifying potential muscle weakness. 

All analyses were conducted by an independent statistician using IBM SPSS Statistics, version 29.0.0.0.241.

## RESULTS

**Figure 1:**
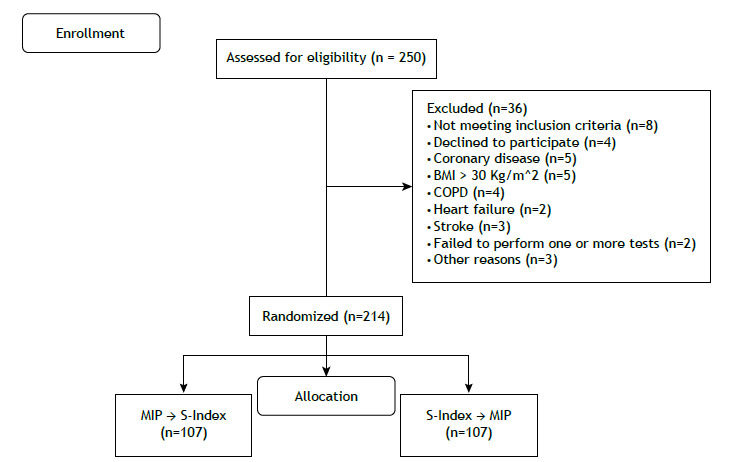
Flowchart of participant recruitment and eligibility. Legend: BMI, body mass index; COPD, chronic obstructive pulmonary disease; S-Index, maximal dynamic inspiratory muscle pressure index; MIP, maximal inspiratory pressure.

Among the 250 volunteers recruited through public invitation, 36 were excluded for reasons detailed in Figure 1. Of the 214 participants evaluated, 107 were male, with ages ranging from 20 to 65 years (mean age: 44.95 ± 14.2 years). Table 1 presents the descriptive data of the participants (additional details are provided in Supplementary Table S1). Demographic and continuous variables were reported as means, standard deviations, medians, and interquartile ranges.

**Table 1:** Demographic and anthropometric data and descriptive statistics of the study sample.

					Shapiro-Wilk		Percentiles	
		Mean	Median	SD	W	p	25th	75th
Age (y)	214	44.95	45.50	14.20	0.93	< 0.001	33.00	58.75
Weight (kg)	214	70.58	70.00	11.23	0.97	< 0.001	62.25	80.00
Height (m)	214	1.66	1.65	0.10	0.93	< 0.001	1.59	1.74
BMI (kg/m^2^)	214	25.45	25.71	3.31	0.88	< 0.001	23.34	28.21
FVC (L)	214	3.71	3.51	0.83	0.97	< 0.001	3.08	4.32
FVC (% pred)	214	96.70	97.05	4.70	0.74	< 0.001	94.80	98.66
FEV_1_ (L)	214	2.78	2.81	0.50	0.99	0.127	2.43	3.09
FEV_1_ (% pred)	214	88.25	89.00	7.72	0.97	< 0.001	81.42	94.46
FEV_1_/FVC (% pred)	214	81.46	81.00	2.16	0.93	< 0.001	80.00	83.00
HGdUL (kg)	214	34.73	32.00	10.48	0.90	< 0.001	28.00	38.00
MIP (cmH_2_O)	214	100.68	97.35	17.03	0.96	< 0.001	86.67	112.00
MEP (cmH_2_O)	214	108.46	102.35	17.03	0.96	< 0.001	90.28	124.00
MIP (% pred)	214	96.66	97.07	6.17	0.93	< 0.001	93.88	100.14
MEP (% pred)	214	94.88	95.20	8.25	0.92	< 0.001	92.34	108.1
S-Index (cmH_2_O)	214	93.33	92.50	16.38	0.98	0.005	80.00	105.00
PIF (L/s)	214	4.65	3.93	6.741	0.21	< 0.001	3.01	4.87
Volume (L)	214	3.48	3.27	0.832	0.97	< 0.001	2.85	4.08
Level of physical activity, IPAQ - SF								
*High (> 3,000 MET-min/week)*	17 (7.9%)							
*Moderate (600-3,000 MET-min/week)*	38 (17.8%)							
*Low (<600 MET-min/week)*	159 (74.3%)							

Age, years; BMI, body mass index; cm, centimeters; FEV_1_, forced expiratory volume in the first second; FVC, forced vital capacity; HGdUL, hand grip of the dominant upper limb; IPAQ, International Physical Activity Questionnaire; kg, kilograms; L: liters; L/s, liter per sec; MET, metabolic equivalent of task; min, minutes; m, meters; PIF, peak inspiratory flow; y, years; S-Index, maximal dynamic inspiratory muscle pressure index; MIP, maximal inspiratory pressure; MEP, maximal expiratory pressure; W, Shapiro-Wilk statistical test.

**Figure 2:**
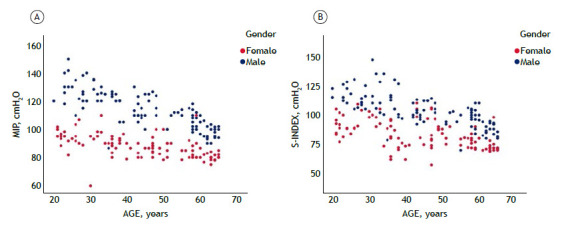
Scatter plots of maximal inspiratory pressure (MIP) (A) and maximal dynamic inspiratory muscle pressure index (S-Index) (B) by sex (females and males) and age group. Legend: MIP, maximal inspiratory pressure; S-Index, maximal dynamic inspiratory muscle pressure index.

There were no significant differences in MIP and S-Index measurements when randomized by evaluation sequence, regardless of the test order. However, the median MIP was significantly higher than the median S-Index (median [IQR]: MIP = 97.2 [96.7-112] vs. S-Index = 92.5 [80-105] cmH_2_O; p<0.001). The mean difference between medians was 8.15 cmH_2_O (95%CI: 7.00-9.18). Both the S-Index and MIP varied significantly by age (p<0.01) and sex (p<0.001), as shown in Figure 2.

The S-Index and MIP were strongly correlated (rho=0.822; 95%CI: 0.770-0.859; p<0.001), as were the S-Index and HGdUL (rho=0.841; 95%CI: 0.760-0.853; p<0.001). In order to assess the variables influencing the S-Index in healthy adults, multiple linear regression analysis was performed using two models. Model 1 included sex and age as independent variables in all MIP prediction equations, whereas Model 2 included sex, age, and handgrip strength measured in the dominant upper limb (HGdUL) to evaluate the extent to which HGdUL improves the prediction of the S-Index in healthy Brazilian adults.

Regression analysis showed that Model 1, which included sex and age, had an R of 0.793 and an R^2^ of 0.629. Model 2, which added handgrip strength (HGdUL), had an R of 0.842 and an R^2^ of 0.708. The inclusion of HGdUL in Model 2 significantly improved the prediction of the S-Index, increasing the model’s explanatory power by 8%. Adjusted R^2^ values were 0.626 for Model 1 and 0.704 for Model 2 (p<0.001 for both comparisons) (Table 2). 

**Table 2:** Linear regression models used to develop reference equations for the maximal dynamic inspiratory muscle pressure index (S-Index).

Model Summary										
Model	R	R^2^	Adjusted R^2^	SE of the Estimate	Change Statistics					Durbin-Watson
					R^2^ Change	F Change	df1	df2	Sig. F Change	
1	0.79^a^	0.63	0.63	10.02	0.63	178.95	2	211	<0.001	
2	0.84^b^	0.71	0.70	8.91	0.080	56.85	1	210	<0.001	1.76
			Coefficients						95% CI	
Model			Predictors	Unstandardized B (Estimates)	Coefficients SE	Standardized Beta	t	Sig.	Lower	Upper
1			(Constant)	107.90	2.37		45.48	<0.001	103.23	112.58
			Age	-0.56	0.048	-0.48	-11.48	<0.001	-0.65	-0.46
			Sex	20.79	1.37	0.64	15.17	<0.001	18.08	23.48
2			(Constant)	69.72	5.49		12.71	<0.001	58.91	80.54
			Age	-0.21	0.063	-0.18	-3.35	<0.001	-0.33	-0.09
			Sex	10.76	1.80	0.33	5.97	<0.001	7.21	14.32
			HGdUL	0.80	0.11	0.51	7.54	<0.001	0.59	1.01

HGdUL, hand grip of the dominant upper limb; R, correlation coefficient; R², coefficient of determination; Adjusted R², adjusted coefficient of determination; SE of the Estimate, standard error of the estimate; R² Change, change in R-squared; F Change, change in F statistic; df1, degrees of freedom 1; df2, degrees of freedom 2; Sig. F Change; significance of the change in F.

According to our study, sex, age, and handgrip strength were significant predictors of the S-Index in healthy Brazilian adults. The regression model was statistically significant, with an R^2^ of 0.708 (F[3,210] = 169.8; p<0.001). The general equation describing the relationship between these variables is Y = B0 + B1×X1 + B2×X2 + B3×X3 + … + Bn×Xn. The proposed equation for men is: S-Index = 69.72 + 10.765×1 - 0.211×age + 0.797×HGdUL. For women, the equation becomes: S-Index = 69.72 - 0.211×age + 0.797×HGdUL. Additionally, the study calculated the LLN for the S-Index across the different age groups, as well as the cut-off values for ventilatory muscle weakness in men and women based on the S-Index Deviation Score (SDS) (Table 3).

**Table 3:** Lower limits of normality (LLNs) for the maximal dynamic inspiratory muscle pressure index (S-Index; cmH_2_O), based on Z-scores and S-Index Deviation Score (SDS) used for cut-off points for ventilatory muscle weakness.

	Sex			
	Female		Male	
	LLN S-Index, cmH_2_O	SDS, cmH_2_O	LLN S-Index, cmH_2_O	SDS, cmH_2_O
20-29 years	62.79	70.85	86.77	96.81
30-39 years	57.05	64.55	84.18	87.17
40-49 years	56.50	58.12	74.99	75.27
50-59 years	50.08	54.14	68.27	72.27
60-65 years	45.24	48.33	60.80	71.94

LLN, lower limit of normality based on the Z-score, calculated using the formula LLN = age-range specific mean − 1.645 standard deviation (SD); S-Index Deviation Score (SDS) reflects the number of standard deviations below the peak mean S-Index and serves as a descriptive and context-appropriate metric for identifying muscle weakness.

The final equation for predicting the S-Index is:

S−Index=69.72(±5.49)+10.765(±1.80)×sex(men=1,women=0)−0.211(±0.06)×age+0.797(±0.11)×HGdUL

Standard Error of the Estimate (SEE) = 8.91; Adjusted R^2^ = 0.704

A Bland-Altman analysis was performed to assess the agreement between the predicted and actual S-Index values. The results demonstrated an adequate level of agreement, with most data points falling within the established limits, indicating that the prediction model reliably estimates inspiratory muscle strength. The Bland-Altman plot is presented in the Supplementary Materials (Supplementary Figure S2).

## DISCUSSION

This study introduces the first sex-specific reference equation for calculating the S-Index (cmH_2_O) in healthy Brazilian adults using a standardized method.^21^ The S-Index test involves maximal, fast, and forceful inspirations from residual volume to total inspiratory capacity,^6^ ^,^ ^7^ and may offer a more functional assessment of inspiratory muscle strength than traditional static measurements, such as MIP, due to its use of dynamic maneuvers. The S-Index was measured using validated PowerBreathe^®^ devices,^5^ ^,^ ^7^ ^,^ ^9^ ^,^ ^10^ ^-^ ^14^ ^,^ ^26^ ^,^ ^27^ and the test-retest reliability was shown to be excellent,^4^ ^,^ ^8^ supporting its application in both healthy individuals and patients.^4^ ^,^ ^5^ ^,^ ^7^ ^,^ ^8^ ^,^ ^10^ ^-^ ^14^

However, few studies have employed a standardized protocol for measuring the S-Index.^6^ ^,^ ^7^ In 2023, Kowalski and Klusiewicz proposed guidelines to minimize methodological variability and provide reliable reference values for this parameter.^21^ In the present study, sex, age, and handgrip strength were identified as significant predictors of S-Index variation. 

Kowalski and Klusiewicz also recently presented reference values for the S-Index in athletes of both sexes aged 18-39 years, reporting mean S-Index values of 70.7 ± 24.1 cmH_2_O for women and 128.7 ± 28.8 cmH_2_O for men in non-athlete populations. Although the age ranges and physical activity profiles differ from those of the current sample, their findings support the influence of age, sex, and physical activity level on S-Index performance, corroborating our results. 

To date, no reference equation for measuring the S-Index in adults has been proposed in Brazil or globally.^27^

The thresholds for identifying respiratory muscle weakness in men and women were based on the S-Index Deviation Score (SDS). This parameter reflects the number of standard deviations below the peak mean S-Index and serves as a context-appropriate metric for identifying muscle weakness. Previous studies have used the T-score to establish normative values ​​and define diagnostic criteria for osteoporosis. Notably, the term “T-score” is specifically defined by the World Health Organization in the context of bone mineral density and refers to the number of standard deviations a measurement is from the mean of a young, healthy reference population, typically for diagnosing osteoporosis.^24^ ^,^ ^25^

In our study, we applied similar statistical reasoning to define a threshold for inspiratory muscle weakness, using a value corresponding to 2.5 standard deviations below the mean S-Index of the youngest adult group (20-29 years). However, we acknowledge that referring to this value as a “T-score” could be misleading outside the context of bone density. Therefore, we adopted the term “S-Index Deviation Score (SDS)” to distinguish it from the conventional T-score. 

We are aware that in the original study by Ana Lista-Paz et al. (2023), the authors applied a T-score threshold of ≥2.5 standard deviations below the mean peak pressure achieved by young adults to establish a single absolute cut-off point for respiratory muscle weakness, separately for men and women.^36^

In contrast, our study adopted a different approach. Rather than determining a single cut-off value, we established a minimum expected value (LLN) for each age group and sex, thereby accounting for physiological variations between men and women as well as the age-related decline in respiratory muscle strength consistently reported in previous studies.^1^ ^,^ ^20^ By calculating age- and sex-specific thresholds, our methodology offers a more precise and clinically relevant assessment of ventilatory muscle weakness. It reflects the natural changes associated with aging rather than applying a fixed value across all adult age groups. This approach improves diagnostic accuracy and aligns with the growing body of literature emphasizing the need for age- and sex-adjusted reference values for respiratory muscle strength.

The study sample reflected the demographic and anthropometric characteristics of healthy Brazilian adults. Results showed that both the S-Index and MIP can be predicted using anthropometric data and handgrip strength across different age groups. The S-Index values were lower than the MIP values, consistent with the fact that isometric forces are typically greater than those generated during isotonic contractions. Both MIP and S-Index assessments are influenced by various factors, including the pressure gauge, interface, air leaks, posture, test instructions, and examiner encouragement, among others.^1^ ^-^ ^3^ ^,^ ^21^ ^,^ ^31^ Variability in these measurements may also stem from differences in reference values proposed for individuals of the same sex and age.^(20, 33-35)^

As mentioned previously, no S-Index reference equations have yet been established for the Brazilian population-a gap this study aimed to address. Using the most widely accepted methodology for MIP reference equations,^20^ the present study proposes a reference equation for the S-Index that aligns with previous predictions of ventilatory muscle function. Age significantly impacted maximal respiratory pressures, and incorporating handgrip strength (HGdUL) enhanced the associations among sex, age, MIP, and the S-Index. The equation explained 70.4% of the S-Index variance (adjusted R² = 0.704) and significantly improved predictive capacity (∆R² = 0.079; p<0.001). 

In order to better understand the S-Index LLN, both Z-scores and the SDS were calculated for different age and sex groups. While Z-scores are useful for interpreting lung function across aging, they may be less suitable for assessing respiratory muscle function, since strength can be preserved or improved through conditioning or training beyond age-related expectations.^24^ ^-^ ^28^ ^,^ ^32^ ^,^ ^37^ Therefore, defining respiratory muscle weakness using age- and sex-specific cut-offs is more appropriate than applying a fixed MIP threshold (e.g., ≤60 cmH_2_O), as often used in systematic reviews of inspiratory muscle training.^39^ ^,^ ^40^ Differences between Z-scores and SDS influence LLN calculation-particularly in older adults, where Z-scores are consistently lower. 

This study has several strengths, including its large, well-matched sample from two centers, with balanced age and sex distributions. Rigorous inclusion and exclusion criteria minimized confounding factors, such as obesity, smoking, lung or neuromuscular conditions, and physical activity levels. Our structured methodology followed the ATS/ERS 2002 protocol-Brazil’s most widely used method for MIP/MEP assessment^1^ ^,^ ^2^-and the standardization procedures by Silva et al.^6^ ^,^ ^7^ ^,^ ^9^ ^,^ ^21^ All evaluators were extensively trained, and strict quality control measures were applied during testing and retesting.^24^ ^,^ ^36^

Although the sample was not fully randomized due to ethical constraints, recruitment strategies such as outreach via social media and community health centers helped reduce bias. Motivational factors affecting MIP and S-Index performance were also mitigated through proper volunteer guidance. 

The analyzed age range (20 to 65 years) may limit the clinical applicability of the reference values-particularly given the aging population and the higher prevalence of respiratory and cardiovascular diseases in individuals over 70 years of age. Nevertheless, the new reference equations and SDS provide improved clinical interpretation of maximal respiratory pressures, helping avoid misdiagnoses of respiratory muscle weakness and refining the selection of candidates for intervention.

Further research is needed to validate these S-Index reference equations in other populations and to establish cut-off values for respiratory muscle weakness in specific patient groups, such as those with COPD, heart failure, or neuromuscular diseases, among others. 

Our study presents the largest dataset of S-Index measurements to date in Brazil, using a standardized methodology aligned with international standards and clinical practice guidelines. We established LLN and S-Index cut-off points for both sexes across different age groups, enabling the identification of respiratory muscle weakness. These findings have significant clinical implications and offer immediate applicability for identifying respiratory muscle weakness and selecting appropriate candidates for targeted training interventions and follow-up. 
